# Association of Broiler Litter Microbiome Composition and *Campylobacter* Isolation

**DOI:** 10.3389/fvets.2021.654927

**Published:** 2021-05-24

**Authors:** Robert Valeris-Chacin, Maria Pieters, Haejin Hwang, Timothy J. Johnson, Randall S. Singer

**Affiliations:** ^1^Department of Veterinary and Biomedical Sciences, College of Veterinary Medicine, University of Minnesota, Saint Paul, MN, United States; ^2^Department of Veterinary Population Medicine, College of Veterinary Medicine, University of Minnesota, Saint Paul, MN, United States; ^3^Veterinary Diagnostic Laboratory, College of Veterinary Medicine, University of Minnesota, Saint Paul, MN, United States

**Keywords:** *Campylobacter*, broilers, litter, microbiome, case-control

## Abstract

Infection with *Campylobacter* species is one of the leading causes of bacterial diarrhea in humans in the US. Chickens, which become colonized on the farm, are important reservoirs of this bacterium. *Campylobacter* can establish itself in the broiler house via a variety of sources, can survive in the litter of the house, and possibly persist over successive flock cycles. However, the role of the broiler litter microbiome on *Campylobacter* persistence is not clear. A matched case-control study was conducted to determine whether the broiler litter microbiome composition was associated with *Campylobacter* isolation within the broiler house. Flocks were classified as cases when either *Campylobacter jejuni* or *Campylobacter coli* was isolated in boot sock samples, or as controls otherwise. Case and control flocks were matched at the broiler house level. Composite broiler litter samples were collected and used for DNA extraction and 16S rRNA gene V4 region sequencing. Reads were processed using the DADA2 pipeline to obtain a table of amplicon sequence variants. Alpha diversity and differential bacterial relative abundance were used as predictors of *Campylobacter* isolation status in conditional logistic regression models adjusting for flock age and sampling season. Beta diversity distances were used as regressors in stratified PERMANOVA with *Campylobacter* isolation status as predictor, and broiler house as stratum. When *Campylobacter* was isolated in boot socks, broiler litter microbiome richness and evenness were lower and higher, respectively, without reaching statistical significance. *Campylobacter* isolation status significantly explained a small proportion of the beta diversity (genus-level Aitchison dissimilarity distance). *Clostridium* and *Anaerostipes* were positively associated with *Campylobacter* isolation status, whereas *Bifidobacterium, Anaerosporobacter*, and *Stenotrophomonas* were negatively associated. Our results suggest the presence of bacterial interactions between *Campylobacter* and the broiler litter microbiome. The negative association of *Campylobacter* with *Bifidobacterium, Anaerosporobacter*, and *Stenotrophomonas* in litter could be potentially exploited as a pre-harvest control strategy.

## Introduction

*Campylobacter* are Gram-negative non-spore forming bacteria with ~31 species and 13 subspecies (1). Several species of *Campylobacter*, primarily *Campylobacter jejuni* and *Campylobacter coli*, are important human pathogens, causing an estimated 1.3 million cases of diarrhea in the US every year (2). The incidence of *Campylobacter* infections in humans seems to be increasing in the US (3). Furthermore, irritable bowel syndrome, reactive arthritis, and Guillain-Barré syndrome have been associated with previous episodes of campylobacteriosis (4). Sources of *Campylobacter* for humans include chicken meat, beef, raw milk, and water (5, 6).

Chickens become colonized with *Campylobacter* on the farm. Although many broiler chicken farms can be negative for *Campylobacter*, once the bacterium is established in the broiler house, the prevalence of colonization in chickens can reach 100% (7, 8). *Campylobacter* predominantly colonizes the chicken cecum, in which it can multiply up to 10^9^ CFU/g of cecal contents (9) without inducing clinical signs. In addition, *Campylobacter* colonization has been shown to increase the translocation of *Escherichia coli* to extraintestinal sites in chickens (10, 11), potentially leading to systemic disease.

Within the broiler house, bedding material, often referred to as litter, is comprised of a substrate (e.g., wood shavings) and chicken excreta (12). Broiler litter is frequently reused across subsequent flocks after windrowing (akin to composting). Windrowing is performed to control poultry pathogens present in the broiler litter (13). However, there is evidence that *Campylobacter* can persist in reused broiler litter (14–16). Therefore, survival of *Campylobacter* in the broiler litter may play an important role in the ecology and colonization of this bacterium in broiler chickens. *Campylobacter* can also enter the broiler house via rodents, flies, darkling beetles, wild birds, cockroaches, or water (17–22).

Broiler litter harbors a microbiome comprised of a collection of bacterial, viral, fungal, and protozoan microorganisms. The bacterial component of the broiler litter microbiome has been shown to commonly include genera associated with the gut and/or the environment. For instance, *Lactobacillus, Escherichia, Bacteroides*, and *Brevibacterium* have been documented in broiler litter and are common members of the gut microbiome, while *Salinicoccus, Arthrobacter*, and *Brachybacterium* are present in both, broiler litter and soil. Furthermore, bacteria such as *Clostridium* and *Corynebacterium*, members of the gut and soil microbiomes, have also been detected in broiler litter (12, 23–27).

The interactions between *Campylobacter* and the bacterial members of the broiler litter microbiome remain unexplored. The aim of this study was to examine the relationship between broiler litter microbiome composition and the detection of *Campylobacter* in the litter of commercial broiler houses. Identifying microorganisms that are either positively or negatively associated with *Campylobacter* detection could lead to potential interventions to prevent or hinder the successful colonization of the broiler house with *Campylobacter*.

## Materials and Methods

### Study Design

A matched case-control study was carried out to evaluate the study objective. The sampling frame consisted of broiler houses enrolled from six large poultry production companies in the US. Enrolled broiler houses were sampled once during the production cycle, between 3 weeks of age and the end of the grow-out period. For this study, samples that were collected from flocks reared consecutively in the same house were used. Two types of samples were obtained: boot socks and composite broiler litter samples. Two boot socks per sampling were collected following previously published procedures (28) and were used for *Campylobacter* isolation. One composite broiler litter sample representative of the house litter was collected per sampling (28) and was used for the microbiome analysis. Briefly, the composite litter samples were collected while walking the house. The composite litter sample included litter from different areas of the house, such as near water lines, feed lines, and along the walls. The primary substrate of the litter was wood shavings; no information regarding litter management was obtained from the participating companies. Flock age and season at the time of sampling were recorded and used in data analysis.

Flocks were classified as cases when either *C. jejuni* or *C. coli* was isolated in boot sock samples or as controls if no *Campylobacter* was isolated. Fifty-one case flocks were individually matched to 139 control flocks by broiler house in a variable case-control ratio (Table 1) for a total of 190 flocks. The case-control assignment was employed to categorize each flock as colonized by *Campylobacter* near the age of slaughter, which has been shown to be correlated with chicken carcass contamination with this bacterium in the processing plant (28). For simplicity, the case-control assignment will be referred to as *Campylobacter* isolation status of the flock.

**Table 1:** Distribution of the ratios used to individually match flocks with isolation of *Campylobacter* (cases) to flocks with no *Campylobacter* isolation (controls).

**Case-control ratio**	***n***	Frequency^*^
1:1	9	23.7
1:5	8	21.1
2:4	4	10.5
1:4	3	7.9
1:6	3	7.9
2:7	3	7.9
1:2	2	5.3
1:3	1	2.6
1:8	1	2.6
3:4	1	2.6
2:2	1	2.6
2:1	1	2.6
3:1	1	2.6

* *Frequency in percentage*.

### Isolation of *Campylobacter*

Boot sock samples were collected and transported overnight to the laboratory at 4°C. Upon arrival, 20 mL Bolton broth (Oxoid, UK) was added to each boot sock and then homogenized. Supernatants were recovered and placed in polyurethane tubes with loose caps (Corning Science, Mexico). Samples were incubated at 42°C for 24 h under microaerophilic conditions (5% O_2_, 10% CO_2_, and 85% N_2_). Afterwards, samples were streaked onto CampyCefex plates (Becton, Dickinson, and Company, USA) supplemented with Preston (Oxoid, UK) and incubated at 42°C for 48 h under microaerophilic conditions. *Campylobacter* colonies were presumptively identified through the observation of typical morphological characteristics (29). Five colonies per plate were selected and streaked for a second passage onto CampyCefex plates supplemented with Preston as performed in the initial step. After a further passage on 5% sheep blood agar (Becton, Dickinson, and Company, USA) to ensure purity, isolates were speciated through PCR.

### Molecular Identification of *Campylobacter* Isolates

DNA from isolates was extracted using proteinase K treatment (0.1 mg/mL) and incubation at 55°C and 80°C for 10 min each in a water bath. Afterwards, 80 μL of DNAse-free water were added to the samples, which were centrifuged for 2 min at 4,500 *g*. Supernatants (with genomic DNA) were harvested and stored at 4°C until use.

Genomic DNA was used for a multiplex PCR employing two primers specific to the *Campylobacter* genus and three primers for discrimination between *C. jejuni* and *C. coli* (30–32). Two microliters of gDNA from presumptive *Campylobacter* isolates were added to 23 μL of PCR reaction mix, which contained 10.25 μL of nuclease-free water, 5 μL of 5X Green GoTaq Flexi buffer (Promega, USA), 3 μL of 25 mM MgCl_2_, 1 μL of 10 mM dNTPs, 0.5 μL of 10 μM lpxA *C. coli* forward primer (5′-AGACAAATAAGAGAGAATCAG-3′), 0.5 μL of 10 μM lpxA *C. jejuni* forward primer (5′-ACAACTTGGTGACGATGTTGTA-3′), 0.5 μL of 30 μM lpxARKK2m reverse primer (5′-CAATCATGDGCDATATGASAATAHGCCAT-3′), 1 μL of 10 μM C412 forward primer (5′-GGATGACACTTTTCGGAGC-3′), 1 μL of 10 μM C1228 reverse primer (5′-CATTGTAGCACGTGTGTC-3′), and 0.25 μL of 5 U/μL GoTaq DNA polymerase (Promega, USA). The following PCR program was used: 5 min at 95°C initially, 30 cycles comprising 30 s of denaturation at 94°C, 30 s of annealing at 50°C, and 1 min of extension at 72°C. The last cycle was followed by 7 min of final extension at 72°C. Amplicons were visualized using UV light after 1.5% agarose gel electrophoresis, with ethidium bromide as DNA stain and 100 bp DNA ladder as standard. The size of the expected products was 816 bp for *Campylobacter* genus, 331 bp for *C. jejuni* and 391 bp for *C. coli*.

### DNA Extraction and 16S rRNA Gene Sequencing

Composite broiler litter samples were prepared by adding PBS (1:1 v/w) and then vortexed for 1 min. Particles present in the solution were allowed to sediment for 5 min, and the supernatant was collected. Supernatant was used for DNA extraction with the PowerSoil kit (Qiagen, Germany) following manufacturer's instructions. The V4 region from 16S rRNA gene was amplified and dual indexed during library preparation (33). Sequencing of the amplicons was performed using MiSeq 2x300 bp platform at the University of Minnesota Genomic Center (UMGC).

### Bioinformatics Analysis

All bioinformatics analyses were performed using R version 4.0.3 (34). The quality profiles of the reads for each sample were visually inspected separately for forward and reverse reads. Primers and adapters were trimmed from all reads (Trim Galore version 0.6.4_dev for adapter removal; URL: http://www.bioinformatics.babraham.ac.uk/projects/trim_galore/). The last 30 bases of forward reads and the last 50 bases of reverse reads were also trimmed. Denoising of reads, merging of paired reads, and removal of chimera were performed using the DADA2 package version 1.18.0 (35). The DADA2-formated training fasta file derived from the Silva Project's version 138 release (36) was used to assign taxonomy to the amplicon sequence variants (ASVs) obtained from the previous steps via a naïve Bayesian classifier (37). Furthermore, exact matching was performed to assign species to ASVs (38). Contaminant ASVs were inferred using the frequency approach of the decontam package version 1.10.0 (39) and pruned from the data set. Amplicon sequence variants from Eukarya, chloroplasts, and mitochondria were removed, as were those present in <1% of the samples. Chao1, Abundance-based Coverage Estimator of species richness (ACE), Fisher, Shannon, and Inverse Simpson alpha diversity indices were computed and used in conditional logistic regression models. Aitchison, Bray-Curtis, and Jaccard beta diversity dissimilarity distances were computed at the ASV-level and used in NMDS and PCoA plots. Additionally, beta diversity dissimilarity distances, calculated at the ASV and genus-level, were used in stratified PERMANOVA (40).

### Statistical Analyses

The association between *Campylobacter* isolation status and alpha diversity was assessed using conditional logistic regression in Stata 16.1 (41). *Campylobacter* isolation status was regressed on each of the alpha diversity indices, adjusting for flock age and sampling season, with broiler house as stratum, and robust standard errors. Multiple imputation to handle missing data on the variable flock age was performed. The association between *Campylobacter* isolation status and beta diversity was evaluated using PERMANOVA stratified by broiler house, with *Campylobacter* isolation status as predictor, and each beta diversity dissimilarity distance as regressor.

Differentially abundant bacteria by *Campylobacter* isolation status were detected with and without aggregation of read counts at the genus level. Read counts were transformed into centered log ratio (42). The feature (genus or ASV, respectively) with the highest centered log ratio was dropped from the data set to avoid collinearity (43). The number of features was reduced using LASSO conditional logistic regression from clogitL1 package version 1.5 in R (44). The regularization parameter (λ) was selected via leave-one-out cross-validation. LASSO selected features were used as predictors in a conditional logistic regression model with *Campylobacter* isolation status as regressor. Adjustment for potential confounders and handling of missing data were performed as indicated for the evaluation of alpha diversity. The significance level was set *a priori* at 0.05.

## Results

**Figure 1:**
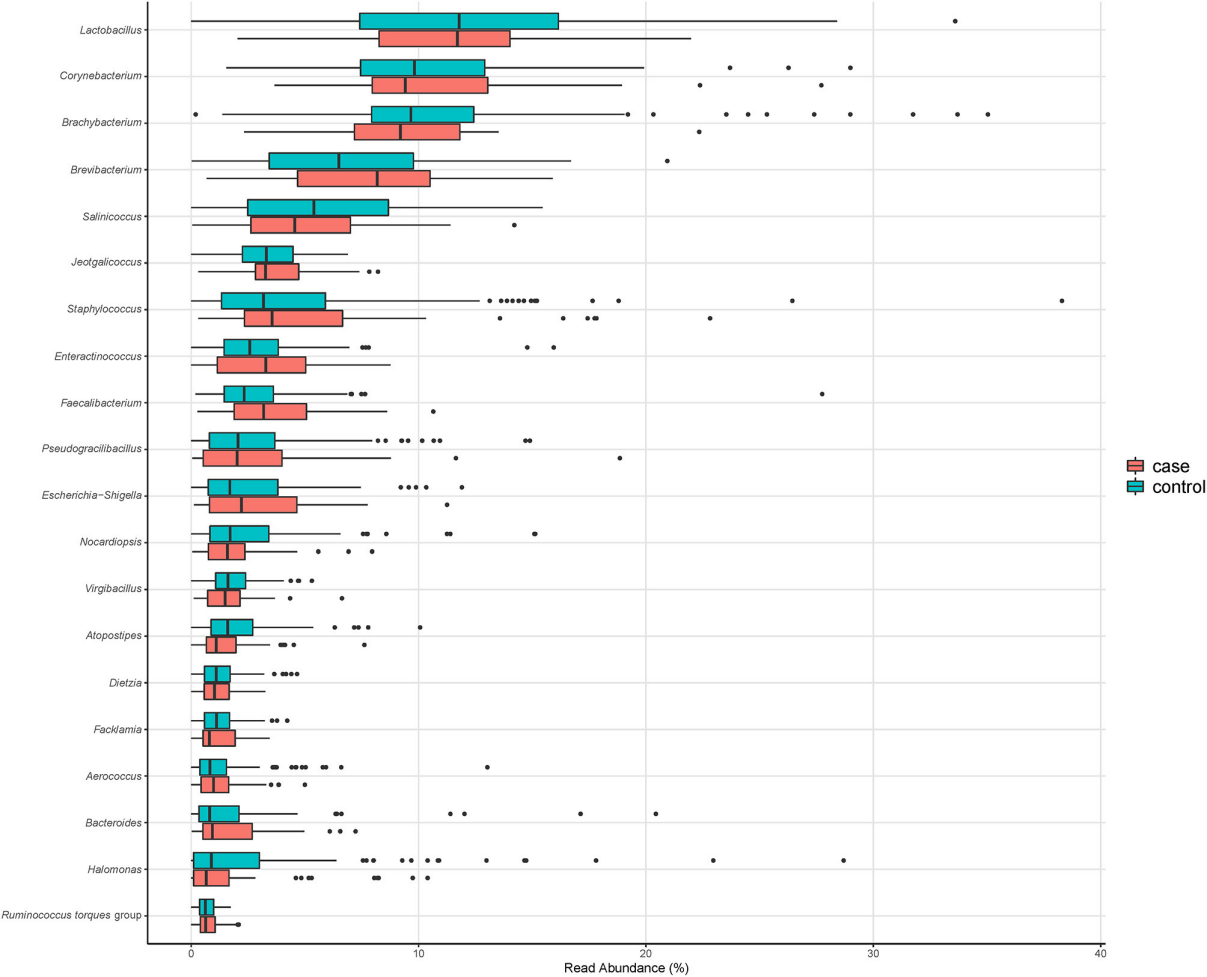
Relative abundance of the 20 most abundant genera in composite broiler litter samples. Samples are separated by the *Campylobacter* isolation status of the flock. Flocks were classified as cases when either *C. jejuni* or *C. coli* was isolated in boot sock samples, or as controls otherwise.

The total number of ASVs detected in the composite broiler litter samples was 2,246. Seventy-three percent of ASVs (1,629) were assigned to 395 genera by the naïve Bayesian classifier. The top five most abundant bacterial genera were *Lactobacillus* (median relative abundance: 11.8%)*, Corynebacterium* (9.8%)*, Brachybacterium* (9.6%), *Brevibacterium* (6.9%), and *Salinicoccus* (5.2%). The 20 most abundant bacterial genera did not change by *Campylobacter* isolation status of the flock; however, their ranks differed (Table 2 and Figure 1).

**Table 2:** Top 20 most abundant bacterial genera in composite broiler litter segregated according to *Campylobacter* isolation status of flocks.

**Rank**	Genera in cases^*^	Genera in controls^*^
1	*Lactobacillus* (11.7)	*Lactobacillus* (11.8)
2	*Corynebacterium* (9.4)	*Corynebacterium* (9.8)
3	*Brachybacterium* (9.2)	*Brachybacterium* (9.7)
4	*Brevibacterium* (8.2)	*Brevibacterium* (6.5)
5	*Salinicoccus* (4.6)	*Salinicoccus* (5.4)
6	*Staphylococcus* (3.6)	*Jeotgalicoccus* (3.3)
7	*Enteractinococcus* (3.3)	*Staphylococcus* (3.2)
8	*Jeotgalicoccus* (3.3)	*Enteractinococcus* (2.6)
9	*Faecalibacterium* (3.2)	*Faecalibacterium* (2.3)
10	*Escherichia-Shigella* (2.2)	*Pseudogracilibacillus* (2.1)
11	*Pseudogracilibacillus* (2)	*Nocardiopsis* (1.7)
12	*Nocardiopsis* (1.6)	*Escherichia-Shigella* (1.7)
13	*Virgibacillus* (1.5)	*Virgibacillus* (1.6)
14	*Atopostipes* (1.1)	*Atopostipes* (1.6)
15	*Dietzia* (1)	*Facklamia* (1.1)
16	*Aerococcus* (1)	*Dietzia* (1.1)
17	*Bacteroides* (0.9)	*Halomonas* (0.9)
18	*Facklamia* (0.8)	*Aerococcus* (0.8)
19	*Alistipes* (0.7)	*Bacteroides* (0.8)
20	*Halomonas* (0.7)	*Ruminococcus torques* group (0.6)

* *Median relative abundance in percentage is given in parentheses*.

### Alpha Diversity

**Figure 2:**
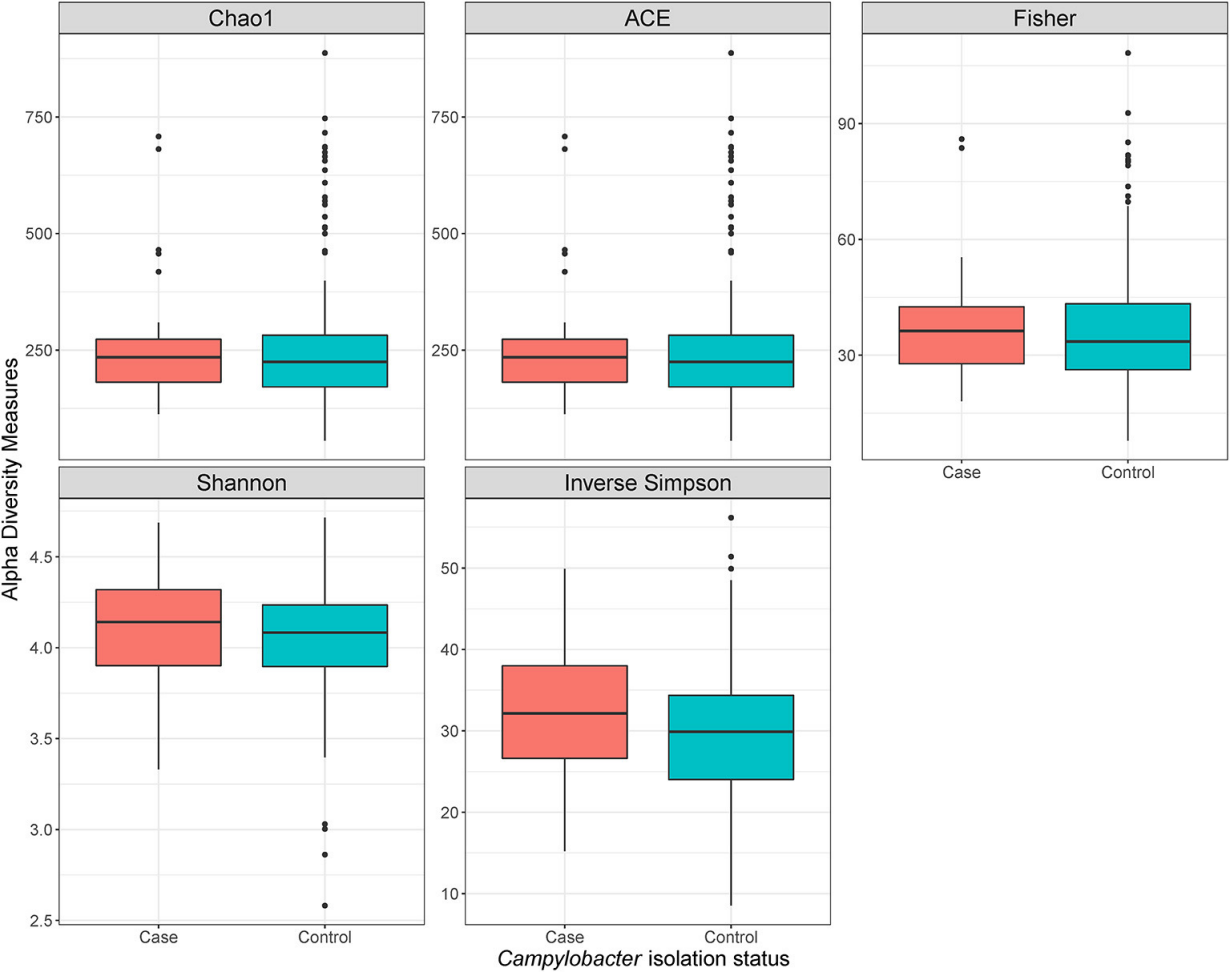
Alpha diversity plots of composite broiler litter samples. Samples are separated by the *Campylobacter* isolation status of the flock. Flocks were classified as cases when either *C. jejuni* or *C. coli* was isolated in boot sock samples, or as controls otherwise.

Using a conditional logistic regression model, it was observed that alpha diversity indices measuring species richness (Chao1, ACE, and Fisher) of composite broiler litter were negatively associated with the odds of *Campylobacter* isolation in boot socks. By contrast, alpha diversity indices estimating species richness and evenness (Inverse Simpson and Shannon) were positively associated with the odds of *Campylobacter* isolation. In other words, fewer bacterial species were detected, on average, in the litter of flocks with *Campylobacter* isolation. The distribution of the species was more homogeneous when compared with litter of flocks without isolation of *Campylobacter* (Figure 2). The associations did not reach statistical significance after adjustment for flock age and sampling season (Table 3).

**Table 3:** Association of alpha diversity indices with *Campylobacter* isolation status of flocks.

**Alpha diversity index**	**aOR**	**95% CI**	***P*-value**
Chao1	0.998	(0.995, 1)	0.151
ACE	0.998	(0.995, 1)	0.151
Fisher	0.98	(0.96, 1)	0.222
Inverse Simpson	1.04	(1, 1.1)	0.067
Shannon	1.9	(0.58, 6.2)	0.291

*aOR, Odds Ratio adjusted for flock age and sampling season, 95%; CI, 95% confidence interval*.

### Beta Diversity

**Figure 3:**
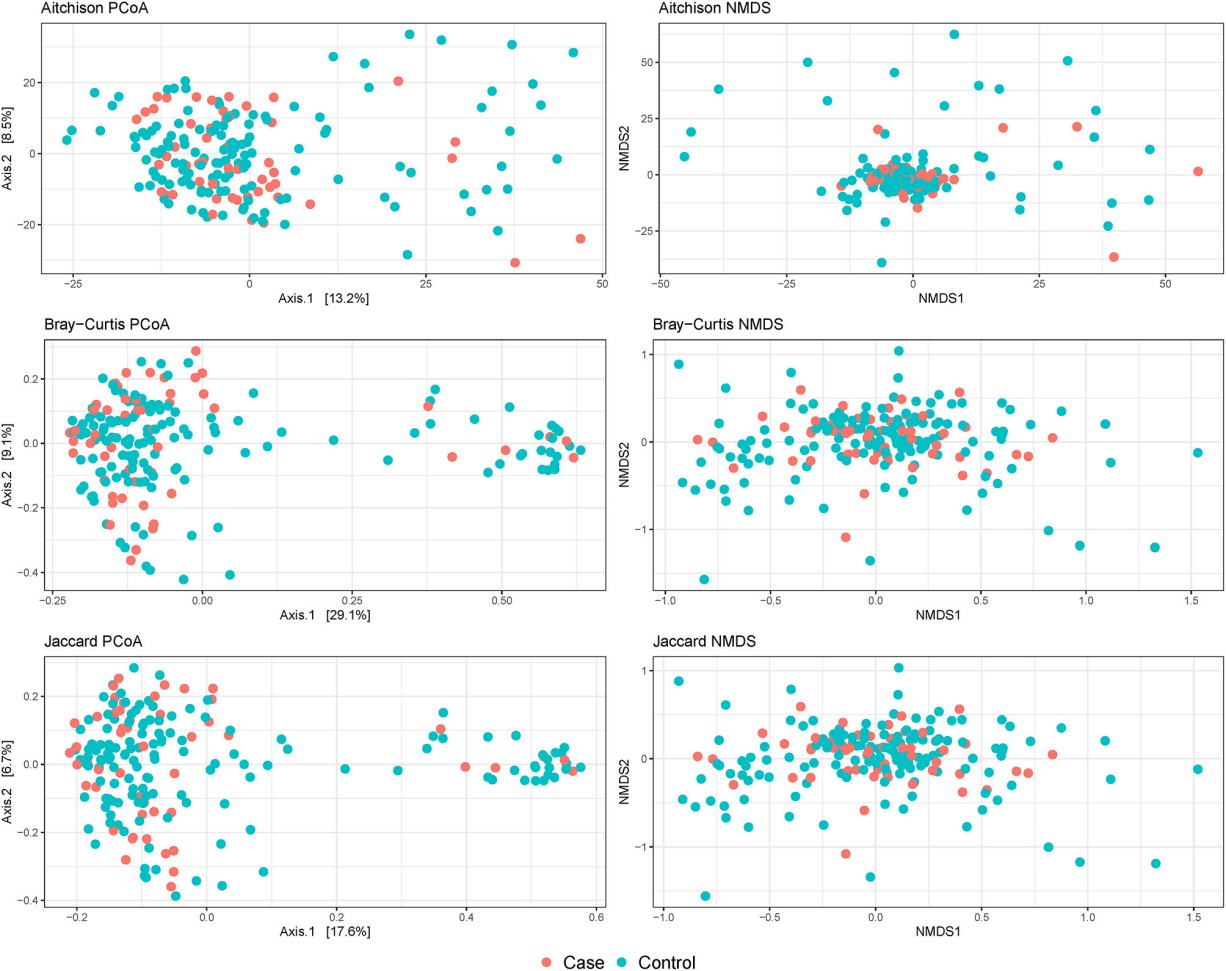
Beta diversity plots of composite broiler litter samples. Samples are colored by the *Campylobacter* isolation status of the flock. Cases (orange) were defined as flocks with isolation of *C. jejuni* or *C. coli* in boot sock samples, and controls (tile) were defined as flocks without isolation of *C. jejuni* or *C. coli* in boot sock samples.

No evidence of distinct clustering of samples according to *Campylobacter* isolation status was observed in any of the evaluated beta diversity dissimilarity distances (Figure 3). Moreover, no statistically significant associations between *Campylobacter* isolation status and beta diversity dissimilarity distances calculated at the ASV level were detected (*P*-value = 0.131, 0.116, and 0.081, for Aitchison, Bray-Curtis, and Jaccard distances, respectively) after stratifying on broiler house to account for the matching. When beta diversity dissimilarity distances were calculated at the genus level, *Campylobacter* isolation status was shown to explain approximately 1% of the variance in Aitchison dissimilarity distance (stratified PERMANOVA: *R*^2^ = 0.007; *P*-value = 0.027). No significant association between *Campylobacter* isolation status and genus-level Bray-Curtis or Jaccard distances (*P*-values = 0.092 and 0.079, respectively) was detected.

### Differentially Abundant Bacteria

**Figure 4:**
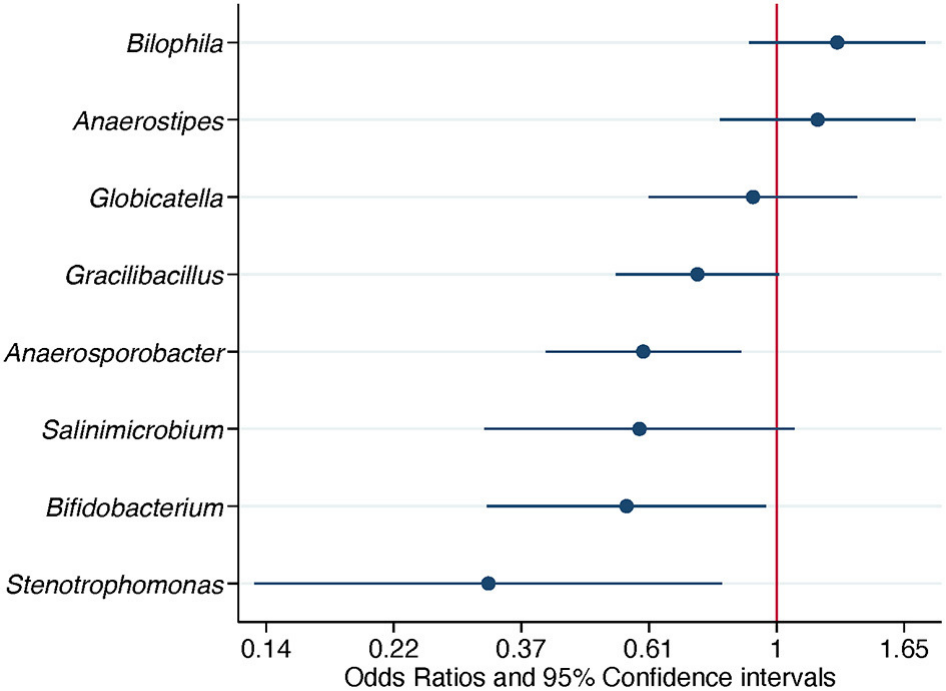
Association of differentially abundant bacterial genera with the isolation of *Campylobacter*. Odds ratios and 95% confidence intervals were obtained from a conditional logistic regression model and adjusted for flock age and sampling season. Differentially abundant bacterial genera were detected in composite litter samples. Log scale was used in the abscissa.

When ASV counts were aggregated at the genus level, 16 genera were selected by the LASSO conditional logistic regression algorithm. Eight out of 16 genera, *Biophila, Anaerostipes, Globicatella, Gracilibacillus, Anaerosporobacter, Salinimicrobium, Bifidobacterium*, and *Stenotrophomonas*, were significantly associated with *Campylobacter* isolation status after adjusting for flock age and sampling season. When these nine genera were evaluated together in a conditional logistic regression model to estimate their independent association with *Campylobacter* isolation status, the relative abundance (transformed as centered log ratio) of *Biophila* and *Anaerostipes* was shown to be positively associated, while the relative abundance of the other bacterial genera was negatively associated. The adjusted ORs (aORs) for *Anaerosporobacter, Bifidobacterium*, and *Stenotrophomonas* reached statistical significance (Table 4 and Figure 4).

**Table 4:** Association of differentially abundant bacterial genera with the isolation of *Campylobacter*.

**Genus**	**aOR**	**95% CI**	***P*-value**
*Bilophila*	1.3	(0.9, 1.8)	0.177
*Anaerostipes*	1.2	(0.8, 1.7)	0.412
*Globicatella*	0.91	(0.61, 1.4)	0.657
*Gracilibacillus*	0.73	(0.53, 1)	0.056
*Anaerosporobacter*	0.59	(0.4, 0.87)	0.007
*Salinimicrobium*	0.58	(0.32, 1.1)	0.083
*Bifidobacterium*	0.56	(0.32, 0.96)	0.035
*Stenotrophomonas*	0.32	(0.13, 0.81)	0.016

*aOR, Odds Ratio adjusted for flock age and sampling season, 95%; CI, 95% confidence interval*.

**Figure 5:**
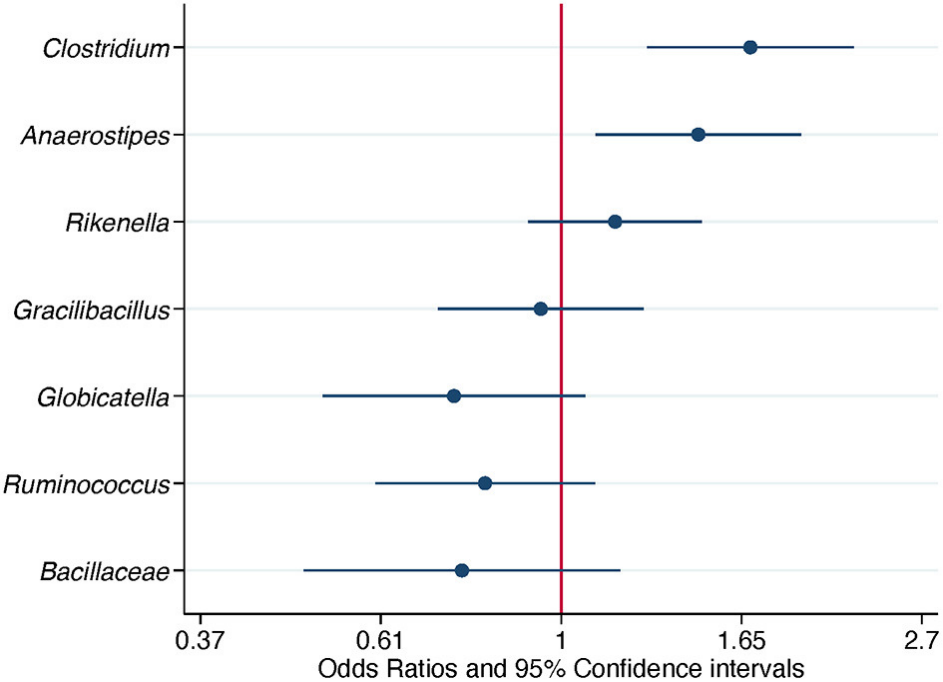
Association of differentially abundant amplicon sequence variants (ASVs) with the isolation of *Campylobacter*. Odds ratios and 95% confidence intervals were obtained from a conditional logistic regression model and adjusted for flock age and sampling season. Differentially abundant ASVs were detected in composite litter samples. The classification at the genus level or at the family level if the former was not obtained, is shown for each ASV. Log scale was used in the abscissa.

When ASV counts were not aggregated at the genus level, 15 ASVs were selected. Seven out of 15 ASVs were further evaluated after detecting a significant association with *Campylobacter* isolation status, adjusting for flock age and sampling season. Three ASVs, belonging to the genera *Clostridium, Anaerostipes*, and *Rikenella*, were positively associated with the odds of *Campylobacter* isolation. The aORs for *Clostridium* and *Anaerostipes* were statistically significant (Table 5 and Figure 5).

**Table 5:** Association of differentially abundant amplicon sequence variants with the isolation of *Campylobacter*.

**Genus**	**Species**	**aOR**	**95% CI**	***P*-value**
*Clostridium*	*perfringens/thermophilus*	1.7	(1.3, 2.3)	< 0.001
*Anaerostipes*		1.5	(1.1, 1.9)	0.009
*Rikenella*		1.2	(0.91, 1.5)	0.222
*Gracilibacillus*	*halotolerans*	0.95	(0.71, 1.3)	0.697
*Globicatella*	*sanguinis/sulfidifaciens*	0.74	(0.52, 1.1)	0.109
*Ruminococcus torques* group		0.81	(0.6, 1.1)	0.174
*Bacillaceae*		0.76	(0.49, 1.2)	0.219

*aOR, Odds Ratio adjusted for flock age and sampling season, 95%; CI, 95% confidence interval*.

Two *Campylobacter* ASVs were detected, one assigned to *C. coli/jejuni* and another to *C. coli/helveticus/jejuni/upsaliensis*. *Campylobacter* relative abundance, both with and without aggregating at the genus level, was significantly associated with *Campylobacter* isolation status after adjusting for flock age and sampling season (genus-level aOR = 2.6, 95% CI = 1.8–3.7, *P*-value < 0.001; ASV-level aOR = 2.5, 95% CI = 1.8–3.5, *P*-value < 0.001). *Campylobacter* relative abundance was considered to be the mediator of the effects of the other members of the litter microbiome on *Campylobacter* isolation status of the flock; thus, it was not included in the final conditional logistic regression models.

## Discussion

This study explored the association of broiler litter microbiome composition and the *Campylobacter* isolation status of the flock. The results of this study showed that the characteristics of the microbiome in broiler litter differed when categorized by the presence or absence of culturable *Campylobacter* in boot socks. Differences in the overall microbiome composition, captured by genus-level Aitchison distance, were significantly associated with the isolation of *Campylobacter*. Moreover, the relative abundance of several bacteria differed depending on the *Campylobacter* isolation status of the flock. *Clostridium* and *Anaerostipes* were significantly increased in abundance whereas *Bifidobacterium, Anaerosporobacter*, and *Stenotrophomonas* were significantly decreased when culturable *Campylobacter* was detected.

Three of the 20 most abundant genera identified in flocks with and without isolation of *Campylobacter* have not been previously reported in broiler litter. However, these three genera have been commonly detected either in the environment, such as soil and plant roots (*Dietzia* and *Pseudogracilibacillus*, respectively) (45, 46) or in the gut of broilers (*Alistipes)* (47), which is not surprising due to the dual nature of broiler litter as a mixture of bedding and chicken feces.

A nuanced association between alpha diversity of the broiler litter microbiome and the isolation of *Campylobacter* was observed, without reaching statistical significance. Alpha diversity captures the richness and evenness of a microbial population, which can be phrased as how many bacterial species coexist and how even their numbers are, respectively. In flocks with *Campylobacter* isolation, broiler litter microbiome richness was lower and evenness was higher than in flocks with no *Campylobacter* isolation. These data suggest that bacterial populations with few species and with a homogeneous composition may provide better support for the growth and viability of *Campylobacter* in broiler litter. Interestingly, our results contrast with those obtained by Bucher et al. (27), who observed that both richness and evenness were increased when litter microcosms were inoculated with *Salmonella* Heidelberg preconditioned in poultry litter extract.

Broiler litter has an important bacterial component originating in the chicken gut (48). Therefore, it is natural to compare the results of this study with those from research focusing on the interplay between *Campylobacter* and the gut microbiome, specifically, the cecal microbiome. Statistically non-significant associations between *C. jejuni* and alpha diversity indices have been documented in ceca from broiler chickens (49–51), with the exception of higher richness in the cecal content of 28-day-old chickens, 14 days after an experimental infection with *C. jejuni* (50).

A significant association was detected between beta diversity of the broiler litter microbiome and the isolation of *Campylobacter*, specifically when genus-level Aitchison dissimilarity distance was used as a beta diversity estimator. Changes in cecum beta diversity have been shown to occur after experimental inoculation of broiler chickens with *C. jejuni* (49–51). Beta diversity summarizes the overall differences between two microbial populations. Specific bacterial species, called differentially abundant bacteria, are responsible for the beta diversity observed between two microbial populations. Therefore, these results suggest that the broiler litter microbiome harbored bacterial species that increased or decreased when *Campylobacter* was isolated in boot socks. In fact, differentially abundant bacterial genera were detected. Besides the expected higher relative abundance of the genus *Campylobacter* in broiler litter when *Campylobacter* was isolated in boot socks*, Clostridium, Anaerostipes, Bifidobacterium, Anaerosporobacter*, and *Stenotrophomonas* relative abundance also significantly differed according to the *Campylobacter* isolation status of the flock.

A positive association was observed between *Campylobacter* isolation status and an ASV matching the 16S rRNA gene V4 region of *Clostridium perfringens* and *Clostridium thermophilus*. The genus *Clostridium* comprises obligate anaerobic endospore-forming Gram-positive bacteria, some of which are major pathogens for humans and domestic animals. The genus is commonly present in broiler feces, litter, and carcasses (52). *Clostridium perfringens* is the causative agent of necrotic enteritis in poultry and has been documented to alter the small intestine microbiome after experimental infection of broilers (53). Skånseng et al. (54) reported a positive correlation of the relative abundance of *C. perfringens* and that of *C. jejuni* in ceca from broiler chickens at slaughter. Similarly, *Clostridium* was shown to contribute to ~9% of the similarity in microbiome composition among *C. jejuni* positive cecal samples (55). However, lack of association between *Clostridium* and *Campylobacter* has also been documented. Froebel et al. (56) reported that *Campylobacter* counts in ceca significantly decreased whereas no significant effect on *C. perfringens* in ileum was observed in broilers after 42 days of prebiotic use. Furthermore, higher *C. perfringens* isolation rates were observed with no significant change in *Campylobacter* prevalence in broiler feces under a drug-free program (57).

An ASV belonging to the genus *Anaerostipes* was positively associated with *Campylobacter* isolation status of the flock. *Anaerostipes* is a genus of endospore-forming bacteria belonging to the family *Lachnospiraceae*. One species, *Anaerostipes butyraticus*, has been shown to be present in broiler chicken ceca and produce short-chain fatty acids (SCFA), specifically butyric acid, which promotes the normal functions of the gut (58). The relative abundance of *Anaerostipes* in the ceca has been shown to increase after the administration of xylo-oligosaccharides (59) and *Bacillus subtilis* (60) to broiler chickens. Connerton et al. (51) documented a negative association between *Campylobacter* and *Anaerostipes* in ceca after experimental infection of broiler chickens at 6 days of age, which contrasts with the positive association observed in broiler litter in this study. A potential explanation for this relationship is that *Campylobacter* might benefit from SCFA produced by *Anaerostipes*, as the utilization of such carbon source has been documented in the chicken gut (10).

The genus *Anaerosporobacter* is another member of the family *Lachnospiraceae*. It was first isolated in soil (61), but is also present in the gut of broiler chickens and pigs (62–64). *Anaerosporobacter* abundance was documented to increase in ceca as a result of the incorporation of enramycin to the feed of broiler chickens throughout a 43-day trial (64) and in feces of broiler chickens with high feed conversion ratio (63). In our study, the genus *Anaerosporobacter* relative abundance was negatively associated with *Campylobacter* isolation, which complements previous findings of the enhancement of the efficacy of a vaccine against *Campylobacter* in chickens by the co-administration of *Anaerosporobacter mobilis* as a probiotic (65).

The abundance of the genus *Stenotrophomonas* decreased when *Campylobacter* was isolated in boot socks. *Stenotrophomonas* are Gram-negative bacteria that are opportunistic pathogens in humans (66), are the predominant bacteria aerosolized from swine feces (67), and have been documented in the poultry environment (68), including litter (12) and poultry meat (69). Li et al. (70) reported that *Stenotrophomonas* was enriched in the jejunum of chickens reared at 33.5°C and was associated with multiple lipid metabolic pathways.

A significant negative association between the genus *Bifidobacterium* and *Campylobacter* isolation status was detected in our study. *Bifidobacterium* are Gram-positive anaerobic bacteria with an extensive arsenal of saccharolytic enzymes and are present in a variety of hosts and environments, including mammals, birds, and insects (71). Several species of *Bifidobacterium* (especially *B. bifidum, B. animalis*, and *B. longum*) are part of commercial probiotic and symbiotic formulations for domestic animals, including poultry (72). *Bifidobacterium* has been shown to competitively exclude *Campylobacter* in broilers. For instance, a one-log_10_ reduction of *C. jejuni* concentration in feces of broilers was observed after a 15-day treatment with *B. longum* in feed (73). Similar results were obtained when combining *B. longum* with the prebiotics galactooligosaccharide (74) and xylooligosaccharide (75).

It is important to highlight that the design of this research was a matched case-control study. Thus, confounders, variables affecting both the isolation of *Campylobacter* in boot socks and the composition of the broiler litter microbiome, need to be adjusted for. Matching on the broiler house accounted for the confounders that vary between broiler houses, for instance, use of probiotics/prebiotics, biosecurity measures, use of organic acids, and other litter treatments (76–79). Flock age and sampling season were adjusted for at the analysis stage as potential confounders. Despite all efforts, confounding might still be present after adjustment (residual confounding), potentially biasing results. In addition, the outcome of this study relied on bacterial isolation, a method with a moderate sensitivity for *Campylobacter* detection that might lead to misclassification of controls. As an example, if *Campylobacter* cells entered a viable not culturable state (16, 80), there is the possibility that flocks were assigned as controls due to lack of *Campylobacter* isolation in boot socks even though *Campylobacter* was present in the broiler house. However, the significant positive association between the relative abundance of *Campylobacter* ASVs in litter and the isolation of *Campylobacter* in boot socks supports the validity of the bacterial isolation approach.

Microbiome studies pose significant challenges, especially during the analysis of differentially abundant bacteria. Observational studies present additional challenges, such as the need for statistical methods that can properly handle highly dimensional compositional data while simultaneously controlling for confounding. In spite of efforts to set guidelines for the statistical analysis of microbiome data (81–84), no clear consensus has been reached in the field. In this study, we selected the centered log relative abundance of the ASVs in a LASSO conditional logistic regression to address the compositionality and the hyperdimensionality of the microbiome data, respectively. The LASSO selected features were included in conditional logistic regression models to estimate their association with the isolation of *Campylobacter* in boot socks. It is important to recognize that this penalized approach might not detect subtle associations with important biological implications. Fortunately, this is an active area of research, and suitable approaches will hopefully be developed and become widely adopted in the near future.

Our results suggest the existence of bacterial interactions between *Campylobacter* and members of the broiler litter microbiome, which may be a reflection of bacterial interactions occurring in the chicken gut. Broiler litter can be considered an environmental extension of the chicken gut since feces are a major component of that litter and chickens display a coprophagic behavior (85). Therefore, it is possible that interventions targeting the broiler litter may have an impact on the chicken gut microbiome. Likewise, it might be the case that probiotics administered to broiler chickens in order to modulate the gut microbiome also have indirect effects when passed on to the broiler litter microbiome through feces. In particular, the negative association of *Campylobacter* with *Bifidobacterium, Anaerosporobacter*, and *Stenotrophomonas* could be potentially exploited using probiotics/prebiotics to reduce the spread of *Campylobacter* in the flock.

This study showed that bacterial interactions related to *Campylobacter* in broiler litter are complex. In order to unravel this complexity and the intricate interrelation between the microbiome of the chicken gut and the broiler litter, future research should assess both microbial communities simultaneously while following flocks under commercial settings. In conclusion, the role of the broiler litter microbiome in the ecology of *Campylobacter* colonization and persistence on-farm needs to be understood to identify and establish effective pre-harvest interventions to control these bacteria. Reducing the risk of campylobacteriosis to consumers due to the application of interventions on the full spectrum of the food system, from farm to fork, will translate into a decrease of the burden that this foodborne disease lays upon the health, welfare, and economy of communities.

## Data Availability Statement

The datasets presented in this study can be found in online repositories. The names of the repository/repositories and accession number(s) can be found below: https://www.ncbi.nlm.nih.gov/, PRJNA686691.

## Author Contributions

RV-C and RS: conceptualization and investigation. RS: funding acquisition. RV-C, HH, and RS: methodology. RV-C, MP, TJ, and RS: formal analysis. RV-C, MP, and RS: visualization. MP and RS: supervision. RV-C and MP: writing—original draft preparation. RV-C, MP, HH, TJ, and RS: writing—review and editing. All authors have read and agreed to the published version of the manuscript.

## Conflict of Interest

The authors declare that the research was conducted in the absence of any commercial or financial relationships that could be construed as a potential conflict of interest.
